# Challenges and Opportunities for Integrating Dealloying Methods into Additive Manufacturing

**DOI:** 10.3390/ma13173706

**Published:** 2020-08-21

**Authors:** A. Chuang, J. Erlebacher

**Affiliations:** Department of Materials Science and Engineering, Johns Hopkins University, Baltimore, MD 21218, USA; achuang8@jhu.edu

**Keywords:** dealloying methods, additive manufacturing, nanoporous metals

## Abstract

The physical architecture of materials plays an integral role in determining material properties and functionality. While many processing techniques now exist for fabricating parts of any shape or size, a couple of techniques have emerged as facile and effective methods for creating unique structures: dealloying and additive manufacturing. This review discusses progress and challenges in the integration of dealloying techniques with the additive manufacturing (AM) platform to take advantage of the material processing capabilities established by each field. These methods are uniquely complementary: not only can we use AM to make nanoporous metals of complex, customized shapes—for instance, with applications in biomedical implants and microfluidics—but dealloying can occur simultaneously during AM to produce unique composite materials with nanoscale features of two interpenetrating phases. We discuss the experimental challenges of implementing these processing methods and how future efforts could be directed to address these difficulties. Our premise is that combining these synergistic techniques offers both new avenues for creating 3D functional materials and new functional materials that cannot be synthesized any other way. Dealloying and AM will continue to grow both independently and together as the materials community realizes the potential of this compelling combination.

## 1. Introduction

The past two decades have seen significant progress in two fields of metallurgy, the dealloying and additive manufacturing of metals. Dealloying is a selective corrosion process that has been extensively developed into a highly effective and facile method for producing self-organized, bicontinuous nanoporous and nanocomposite metals. Additive manufacturing (AM) has revolutionized the design and production of customized metal parts by employing layer-by-layer construction, and the development of AM continues to advance rapidly as commercial industries adopt this emerging technology.

The development of dealloying and that of additive manufacturing have paralleled one another. While aspects of both technologies emerged much earlier, both dealloying and additive manufacturing techniques did not begin to see widespread popularity until the early-to-mid 2000s. In the early 2000s, a series of publications identified the fundamental kinetic processes leading to porosity evolution in dealloying, leading to the identification of many new materials systems that could be processed in this way. In turn, these inspired studies on the mechanical properties and catalytic properties of nanoporous materials. That same decade also saw explosive growth in the additive manufacturing sector owing to the production of low-cost AM units and the development of more user-friendly 3D modeling software. These innovations enabled adoption by not only the manufacturing industry but also the general public [1]. Since this time, these two fields have shown rapid and significant progress from nascent beginnings in basic research to widespread implementation in many applications.

We argue here that AM and dealloying are inherently and uniquely complementary: not only can we use AM to make nanoporous metals of complex, customized shapes, but dealloying can occur simultaneously during AM to produce unique composite materials with nanoscale features of two interpenetrating phases. Such complementarity in materials processing will undoubtedly lead to many fascinating and impactful advances.

## 2. Dealloying

**Figure 1:**
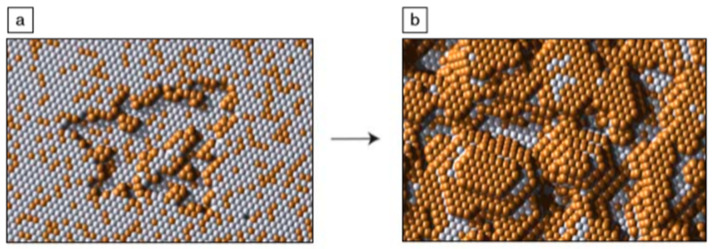
This atomic-scale kinetic Monte Carlo simulation of a dealloying Au-Ag alloy provides a visual model of early porosity evolution during dealloying. (**a**) When the alloy is exposed to the dealloying medium, Ag atoms begin to dissolve away from the surface. Au atoms accumulate at the step edges via surface diffusion processes. (**b**) As more Ag atoms are exposed and removed, the steps break up into islands of Au atoms. Eventually, these islands are undercut, and Au atoms nucleate new mounds, forming the nanoporous structure observed in dealloyed materials. Adapted from [3]. Copyright 2009 by Materials Research Society.

**Figure 2:**
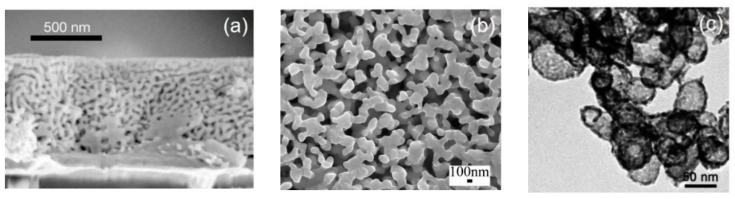
Examples of electrochemically dealloyed nanoporous metals. (**a**) Cross-sectional view of dealloyed Au_32_Ag_68_ thin film. Adapted from [6]. Copyright 2001 by Springer Nature. (**b**) SEM image showing the plane view of Al_40_Cu_60_ post-dealloying in a dilute HCl solution. Adapted with permission from [7]. Copyright 2009 by American Chemical Society. (**c**) TEM image of nanotubular silver formed via dealloying and then reacting with a PdCl_4_^2−^ solution. Adapted with permission from [8]. Copyright 2010 by WILEY-VCH Verlag GmbH & Co.

Dealloying was once only considered a detrimental corrosion process, most commonly as the dezincification of brass alloys, but the field has now grown to encompass an array of processing techniques that take advantage of this corrosion process to make self-organized nanoporous metal networks with extremely high surface areas [2,3]. These techniques have enabled materials scientists to produce topologically complex metals via the selective removal of a sacrificial element from an initially homogeneous precursor alloy. In general, porosity forms during dealloying when the selective dissolution of one element from a multi-component precursor occurs under conditions in which the remaining element reorganizes itself into a porous structure. Such reorganization often occurs by diffusion along the growing interface between the precursor material and the dealloying medium. Figure 1 shows a model of the generic character of the dealloying process and how porosity evolves. Figure 2 shows micrographs of various dealloyed metals, to give a sense of the kind of microstructure formed. Dealloyed microstructures often look like bicontinuous microstructures associated with spinodal decomposition, with pore and ligament diameters in the order of a few tens of nanometers, i.e., with high surface areas not possible to produce by simply loosely sintering powders. Perhaps more importantly, because microstructure evolution occurs by interfacial diffusion, there is no mechanism for creating grain boundaries (with the exception of when dealloying occurs simultaneously with a phase change [4]). Thus, the morphology intrinsically includes geometric saddle points and high step-edge densities [5], which is often useful for catalysis, and in addition, the lack of grain boundaries often leads to materials with better mechanical properties compared to those of porous materials made simply by sintering powders.

### 2.1. Dealloying Techniques

**Figure 3:**
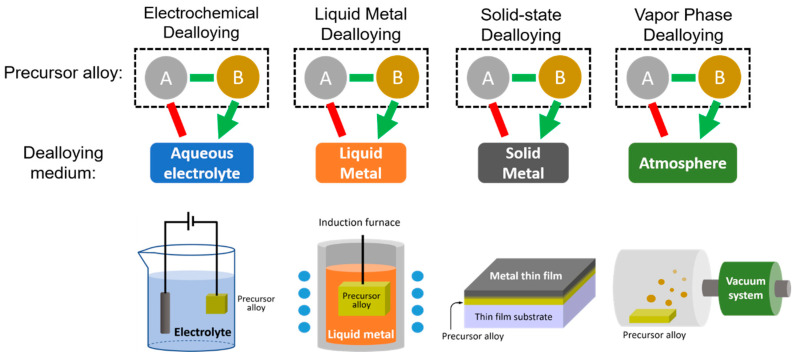
A graphical summary of the four dealloying techniques discussed in the text and the various pathways of dissolution into the four different dealloying mediums.

The variety of dealloying methods has grown as researchers have identified new dealloying media beyond aqueous acidic solutions. Because selective dissolution is driven by some difference in how the elements of the precursor alloy interact with the dealloying medium, changing the dealloying medium changes the driving force behind the dealloying. With more dealloying pathways comes a greater number of compatible material systems, resulting in new nanoporous metals that were previously inaccessible via dealloying. Figure 3 provides a visual summary of the four dealloying techniques discussed in this section. As shown, each technique starts with a precursor alloy of element A and B, but the dealloying medium used in each technique differentiates one method from another. Only element B will dissolve into the chosen dealloying medium, leaving element A behind. The dissolution mechanism also varies by method, but in all cases, the mechanism will selectively remove element B from the precursor alloy. Schematic representations of a typical setup for each technique are also given.

#### 2.1.1. Electrochemical Dealloying

While many variations of dealloying now exist today, electrochemical dealloying was the earliest form of dealloying that attracted attention from the community for the synthesis of nanostructured materials [9,10]. As the name implies, electrochemical dealloying relies on the difference in standard reduction potentials between the two elements of the precursor alloy and the chemistry of the elements in the electrolyte used as the dealloying medium. For example, silver can be selectively dissolved from Ag-rich Ag-Au alloys to form nanoporous Au because Ag is much more easily oxidized than Au. Other examples of nanoporous materials made by electrochemical dealloying include nanoporous precious metals, such as Pt [11], Pd [12] and Ag [8], and other relatively noble metals, such as Cu [7,13] and Ni [14]. The underlying kinetics of electrochemical dealloying were not fully elucidated until 2001 when Erlebacher et al. published a new model for porosity evolution that successfully predicted the characteristic length scale, the average distance between ligaments, of the nanoporous structure [6]. In this model, as the less-noble dissolving element is stripped from the surface, atoms of the remaining more-noble element coalesce into clusters, exposing areas of undealloyed material to the electrolyte. Competition between the rates of these two mechanisms, the rate of dissolution and the rate of interface diffusion, results in the formation of a stable porous structure and governs the characteristic length scale of the porosity. Experimental factors can be adjusted to tune the size of the pores. For example, the rates of both mechanisms increase at a higher applied potential, but because the dissolution rate increases much faster, the characteristic length scale becomes smaller and the resulting porous structure is much finer [15].

#### 2.1.2. Liquid Metal Dealloying

Liquid metal dealloying, unlike electrochemical dealloying, utilizes the difference in enthalpies of mixing between each element of the precursor alloy with a molten third element, which will act as a liquid metal solvent. The third element is chosen such that, at the dealloying temperature, the sacrificial element will dissolve out when the precursor alloy is immersed in a molten bath of the third element, due to a positive enthalpy of mixing between the two elements. As the dissolving species is removed from the precursor, the remaining element reorganizes into a bicontinuous porous structure. This phenomenon was first discovered by Harrison and Wagner in 1959 [16] and was re-introduced by Wada et al. in 2011 [17,18]. Since then, considerable literature has been published on the kinetics of the process and the application of this technique to dealloy materials that cannot be dealloyed electrochemically [19,20], for instance, Ti and refractory metals, which were previously inaccessible due to their relatively low surface mobilities [21]. To date, liquid metal dealloying has been used to make bicontinuous porous structures of Nb [22,23,24], Ti [25], C [26], stainless steel [27] and Co-based alloys [28,29]. Liquid metal dealloyed porous metals combine the electrical conductivity and wear resistance of their bulk counterparts with the high surface area of nanostructured materials and thus are envisioned for many applications in capacitors [22] and batteries [30].

#### 2.1.3. Solid-State Dealloying

Alternatively referred to as solid metal dealloying or solid-state interfacial dealloying, solid-state dealloying is the selective removal of one element from the precursor alloy via solid-state diffusional mechanisms into a metal solvent, forming a bicontinuous microstructure, again resulting from the relative rates of dissolution and interface reorganization. Like for liquid metal dealloying, the driving force behind solid-state dealloying is the difference in enthalpies of mixing between the elements of the precursor alloy and the dealloying medium, leading to selective dissolution. The transport kinetics in solid-state dealloying are generally slower by a few orders of magnitude compared to those in both electrochemical and liquid metal dealloying; however, for the same reason, the ligament size of the dealloyed material tends to be much finer. As such, solid-state dealloying has, to date, been limited to thin film geometries or as an interfacial effect. Wada et al. first reported this technique in 2016, using solid-state dealloying to create a dealloyed interfacial region between the two layers of a diffusion couple [31]. They proposed solid-state dealloying as a new strategy to suppress coarsening during the formation of the porous structure. Coarsening is mediated by thermally activated atomic diffusion at the interface between the precursor and the solvent; thus, operating at a lower temperature can slow coarsening, resulting in a finer morphology with greater surface area [32]. Further work has successfully demonstrated how solid-state dealloying can be applied to thin films, either with a precursor thin film deposited on a solid metal solvent substrate [33] or with both the precursor and the dealloying medium in thin film form [34].

#### 2.1.4. Vapor Phase Dealloying

In the latest variation of dealloying to be carefully elucidated—vapor phase dealloying—the difference between the vapor pressures of the two precursor alloy elements is used to drive the sublimation of one element at an elevated temperature, while the remaining element forms a porous metal structure [35]. The early beginnings of this technique can be found in the vacuum dezincification of brass, which was known to result in porosity [36]. In the 1950s, Balluffi and Alexander demonstrated that the formation of porosity under vacuum is the same process by which Kirkendall voids form in diffusion couples, clarifying the conditions for porosity evolution [36]. This process was recently re-introduced as “vacuum dealloying” by Sun et al. as a new method for preparing porous copper powder from commercial brass powder [37]. In 2018, Lu et al. modified the process to be more environmentally friendly by demonstrating how the evaporated element could be fully recovered using a cold trap during vapor phase dealloying [38]. In addition to the ability to recycle metals from the precursor alloy, this dealloying method also eliminates the etching step and resulting chemical waste produced in other dealloying methods. Due to these advantages, vapor phase dealloying has a great deal of potential as a facile and environmentally friendly technique for producing nanoporous materials. To date, vapor phase dealloying has been limited to Zn-based systems, as Zn has a saturated vapor pressure an order of magnitude higher than those of its alloying elements, such as Ni and Co [35,38]. Further work will be required to apply the environmental advantages of vapor phase dealloying to more alloys.

### 2.2. Applications of Dealloyed Materials

The recent introduction of these new techniques has increased the compositional diversity of dealloyed nanoporous metals and nanocomposites. All of these dealloying methods, each with their own advantages and limitations, can be used together with additive manufacturing (AM) to gain further control over the final shape and microstructure of materials in AM parts, which then, in turn, affect material properties and potential use. Because of their high surface area and relative structural stability, dealloyed materials by themselves have already proven to be incredibly versatile across several industries. Here, we discuss three categories of dealloyed materials’ applications that could benefit from the integration of AM into their processing: (1) electrochemically active materials such as electrocatalysts for fuel cells, battery electrodes or electrochemical actuators; (2) structural materials; and (3) substrates for biomedical applications such as implant surfaces.

Some of the earliest work on dealloyed nanoporous metals evaluated their electrocatalytic properties [39,40]. Like nanoparticles, electrochemically dealloyed nanoporous metals have a high surface-area-to-volume ratio but offer enhanced long-term durability by avoiding the issue of nanoparticle aggregation [41]. Nanoporous Au has a relatively inert catalytic surface but is a good candidate for CO oxidation [42]. Nanoporous Pt-based alloys and Pt-coated materials have also attracted much attention as potential oxygen reduction reaction (ORR) catalysts in proton exchange membrane fuel cells (PEMFCs) [8,43,44]. While much of the energy technologies research on dealloyed materials has focused on noble metals such as Au and Pt, liquid metal dealloyed nanoporous Si has also been reported as a promising new material for Li-ion battery anodes, as the nanoporous structure allows for volume expansion to accommodate repeated lithiation and de-lithiation cycles. Wada et al. reported a higher gravimetric capacity and increased cycle life for nanoporous Si as compared to those for commercial nanoparticle Si [30]. In these examples and for catalysts in general, performance is directly tied to structure. AM could further improve the functionality of these devices by enabling the fabrication of more complex catalytic structures with hierarchical networks of additional internal channels to promote and direct mass transport.

The introduction of liquid metal dealloying and solid-state dealloying techniques opened the door to a greater variety of applications for nanoporous metals by increasing the compositional range of dealloyed materials to include non-noble metals, such as refractory metals, which are defined by their resistance to high temperatures and mechanical wear. Because the dealloying solvent is metal, these two dealloying methods can be used to create high-strength metal–metal nanocomposites. Liquid metal dealloying has already been demonstrated as an effective and facile method for making nanocomposites with remarkable strength and toughness [45]. However, because the rate of the reaction is limited by mass transport in the solvent, this process is difficult to scale up. Laser-based AM provides an opportunity to produce net-shaped dealloyed nanocomposites in one step. Selective melting or sintering, in addition to fusing together powder particles, can also trigger a liquid metal or solid-state dealloying reaction. This strategy is discussed in detail in Section 4.3 of this review.

Dealloyed materials have also demonstrated potential for use in next-generation biomedical implants. The design criteria for biomedical implants are often rigorous and require tailored material properties, which can be provided through dealloying. For example, orthopedic implant materials must exhibit a mechanical response similar to that of bone. A mismatch in stiffness results a disproportional load distribution between the bone and the adjoining implant, which can lead to bone regeneration and implant failure [46,47,48]. Titanium alloys are a good candidate for orthopedic implants due to their biocompatibility and corrosion resistance but are substantially stiffer than bone [48,49,50]. Okulov et al. published a series of studies on using dealloying techniques to tune the mechanical properties of nanoporous Ti and Ti-based nanocomposites to match the elastic behavior of human bone [51,52,53,54]. Some implants are required to be bioresorbable for applications where only temporary support from the implant is needed. Constructing an implant that will provide both adequate mechanical support upon implantation and degrade quickly enough to transfer the load to surrounding tissue is difficult, but one promising solution is to use metal scaffolds with significant porosity. Heidan and colleagues saw increased cell attachment on the nanoporous surfaces of a dealloyed Fe-Mn scaffold and measured an improved degradation rate due to the increased surface area for medium attack [55]. AM has already been demonstrated as an effective process for printing customized implants [56] and incorporating dealloying into the production process will enable the production of nanoporous and nanocomposite biomedical implants with the necessary material properties to improve performance and patient outcomes.

## 3. Additive Manufacturing

The term “additive manufacturing” was formally established in 2009 by the ASTM (formerly known as the American Society for Testing and Materials) F42 Technical Committee to define a new class of manufacturing processes that employed layer-by-layer deposition to print components from 3D model data [1]. This definition sets AM apart from more traditional manufacturing methods, including subtractive methods, such as milling or turning, and formative processes, such as forging and casting. AM has revolutionized the manufacturing industry by substantially increasing the ease and speed of producing prototypes and customized shapes. In additive manufacturing, costs no longer scale with complexity, which means AM can be used to quickly produce cost-effective customized, patient-specific implants [56]. Biomedical implants have been fabricated with a variety of AM techniques including electron beam melting [57], selective laser melting [58], and binder jetting [59]. Liang et al. used multi-jet printing technologies to print custom artificial teeth using scan data of the extracted original teeth [60]. AM has also enabled the manufacturing of components with intricate inner channels and valves, which previously required manufacturing multiple components and welding them together. In building GE’s new LEAP (Leading Edge Aviation Propulsion) engine fuel nozzle, incorporating powder bed fusion AM into the manufacturing process allowed the engineers to redesign the nozzle as a single part, eliminating the previously labor-intensive step of assembling and welding 20 individual parts [61].

However, 3D printed metal components, unlike like their conventionally machined counterparts, commonly exhibit micro-porosity and anisotropic fracture behavior [62]. Usually, anisotropy and porosity are features that are considered problematic, but that is true only in uncontrolled processing. For stents and implants, controlled nanoporosity affords the ability to load the part with drug-eluting functionality, allowing stents or implants to control inflammation as the biological system in which they are put acclimates to them [63]. For parts with mechanical functionality, engineered anisotropy can lead to a much more robust response to applied forces [64]. These observations form the link between AM and dealloying—engineered porosity can be generated in part by electrochemically dealloying the part post-fabrication; even more exciting is the potential to control the thermal history of an AM part by mixing two-component powders, one component of which acts as the dealloying medium (the other component is to be dealloyed). The microstructure can be controlled by the engineered thermal history of the laser-material interaction during AM. This is a challenging problem, as even single-component powders exhibit extremely complex solidification behavior during AM.

Several methods of AM currently exist, the majority of which fall into one of the seven categories of AM processes defined by the ASTM F42 Technical Committee [65,66]. Here, we will discuss two of the most popular AM processes that have potential for use in conjunction with dealloying: direct ink writing (classified as material jetting) and selective laser melting/sintering (both classified as powder bed fusion). Both methods are suited for manufacturing metal components, a basic requirement for combined usage with dealloying, but these two techniques have been identified in particular due to their versatility in processing a wide spectrum of elemental metal and metal alloy materials.

### 3.1. Direct Ink Writing

**Figure 4:**
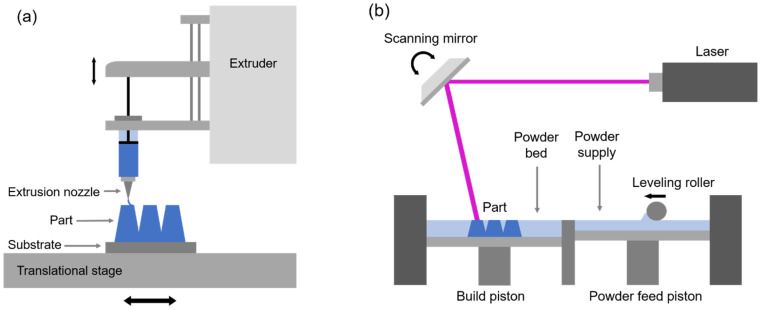
Illustrative depictions of common additive manufacturing processes: (**a**) Direct ink writing (DIW) utilizes a nozzle to deposit ink layer-by-layer at a controlled flow rate in a pattern defined by the digital model. (**b**) Selective laser melting (SLM) works with a bed of powders, instead of ink, by using a laser to selectively heat and fuse together areas of the powder bed to form a solid 3D shape. A fresh layer of powders is distributed by the roller between scans.

Direct ink writing (DIW) describes a class of droplet-based 3D printing processes. An ink deposition nozzle generates a pattern onto the substrate, which sits on a computer-controlled translational stage [67]. Figure 4a shows a simplified diagram of this AM technique. Ceramic, metallic and carbon-based inks have been developed, growing the possibilities for DIW processes to be included in production in a broad diversity of technologies. Additively manufacturing metal components using this method generally requires the use of composite inks consisting of metal nano-/micro-particles suspended in an aqueous organic binder solution [68]. The final printed component of unbonded metallic powders and binder is considered a “green body” and must undergo post-processing to remove the binder from the final material and densify the material. DIW is relatively accessible compared to other AM methods and has been used to print functional devices such as piezo-composites with interpenetrating bicontinuous ferroelectric and polymer phases [69] and complex hybrid metallic structures for batteries and medical implants [70].

### 3.2. Selective Laser Melting/Sintering

The most promising metal powder bed fusion technique currently is selective laser melting (SLM), in which a high-power laser is used to fuse metal powders together into a solid part [71]. Inside the build chamber, a thin layer of loose metal powder is swept onto a substrate by a roller, and then the laser selectively melts and fuses areas according to a digital model. The platform is lowered, a new layer of metal powder is swept onto the surface, and the laser scans the next layer of the build. The process is repeated until the component has been fully built. A schematic of a basic SLM setup is given in Figure 4b. Selective laser sintering (SLS) is a similar method where the laser only sinters the loose powder together instead of completely melting the material. However, this method often leads to unfavorable and uncontrolled porosity within the material, negatively impacting the strength of the final manufactured component [62]. The main advantage of using selective laser melting or sintering is its comparatively wide range of compatible materials, such as Al-based alloys [72], Fe-based alloys [73], Ti-based alloys [74], and metal composites [75,76], in contrast to other powder bed fusion techniques, such as electron beam melting, which is constrained to a far more limited set of materials than SLM or SLS [77].

## 4. Dealloying and Additive Manufacturing

Some progress has already been made in using dealloying and AM methods together for the controlled formation of hierarchical porous structures, but this work can be expanded upon. There are also new pathways that can be opened with the combination of these two types of material processing; while the existing literature has focused on employing dealloying techniques as a post-processing treatment for 3D printed structures, here, we introduce the possibility of using laser-based AM to induce porosity formation during the printing process such that the material is already dealloyed before it leaves the build chamber. While rapid solidification during SLM introduces new complications to the dealloying process, it also provides a vision for a one-step method to produce metal nanocomposites with the material property advantages of a dealloyed microstructure coupled with the manufacturing flexibility of 3D printing. We will then discuss how further processing can produce nano/micro-porous powders from AM nanocomposites.

### 4.1. Porous Materials with Hierarchical Porosity

Interest in hierarchical porous structures stems from their potential applications in microfluidics and catalysis, where a large surface-area-to-volume ratio and fast mass exchange are both necessary for optimal performance [78,79]. Examples of hierarchical porous structures abound in nature. The structures in coral, our own vasculature and our manmade transportation networks are all hierarchical frameworks that facilitate transport through channels of varying length scales. The transportation of fluid, nutrients or people at certain flow rates all depend on a well-designed hierarchical structure. With recent developments in dealloying and additive manufacturing, we can create new hierarchical porous metal networks with better ease and speed, as well as a greater degree of control over the structures at each length scale.

A number of traditional methods exist for fabricating hierarchical porous metallic materials—templating [80], electrodeposition [81] and multistep dealloying [82,83]. However, all these methods are limited in their material flexibility, range of pore sizes and/or control over shape geometry [84]. Additive manufacturing methods, such as direct ink writing and selective laser melting, allow digital control over the design of the macroporous network, meaning researchers can use computer-aided design (CAD) software to design a network to optimize the mass flow and accessibility of the surface sites within the material. AM by itself can also be used to print metal networks with hierarchical porosity, but the resolution of most powder-based AM systems today is limited to tens of microns [85]. Dealloying provides microstructural control of the material’s architecture down to the nanometer scale, translating to an enhanced specific surface area of up to 184.8 m^2^/g in nanoporous graphite, for example [26]. Bulk nanoporous dealloyed materials, however, suffer from sluggish transport through their bicontinuous network of nanosized channels, so introducing structural hierarchy significantly improves mass transport efficiency. The combination of these two complementary materials techniques has the potential to enable rapid transport through a nanoporous structure, increasing the accessibility without sacrificing specific surface area. The synergistic relationship between dealloying and additive manufacturing provides a unique opportunity to develop a simple, rapid fabrication method for hierarchical porous metals whose features can be tuned down to the nanoscale.

**Figure 5:**
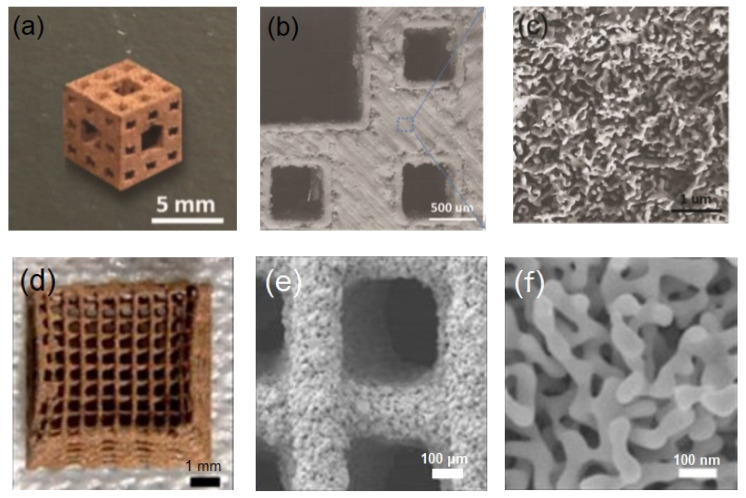
Examples of additively manufactured and dealloyed metals, one of copper (top row) and one of gold (bottom row). Each series of images shows the hierarchical porosity of the sample at increasing magnification, showing three distinct levels of porosity in each sample. (**a**) Macroscale architecture Cu-Mn sample printed via selective laser melting (SLM), with Mn removed via post-additive manufacturing (AM) dealloying (**b**) Microscale gaps between tracks due to imperfect overlaps provide an intermediate level of porosity. (**c**) Self-organized copper ligaments from dealloying. Images (**a**–**c**) are adapted with permission from [74]. Copyright 2019 by WILEY-VCH Verlag GmbH & Co. (**d**) Woodpile-like macroscale architecture of Au-Ag printed via direct ink writing (DIW), with Ag removed via post-AM dealloying. (**e**) Microscale porosity due to degradation of binder from the ink. (**f**) Nanoporous Au structure formed during dealloying. Images (**d**–**f**) are adapted with permission from [73]. Copyright 2018, the authors.

This approach was examined in Song et al.’s review on hierarchical porous metal structures [84], but at the time, no experimental studies had been published on dealloying additively manufactured parts to obtain macroporous structures with nanoporous surfaces. The authors attributed the lack of progress in this direction to the difficulty of obtaining powders of the precursor alloys. Since then, Zhu et al. have demonstrated the feasibility of creating a hierarchical porous gold structure via sequential additive manufacturing and then dealloying without the use of precursor alloy powders [86]. Instead, they used an ink comprised of Au and Ag microparticles combined with a polymeric binder to print the sample via direct ink writing. Afterward, a post-printing heat treatment was used to both remove the binder phase from the green body and allow the Au and Ag atoms to inter-diffuse, creating a homogenous alloy suitable for dealloying. After dealloying, the result was a hierarchical nanoporous Au structure with multiple distinct characteristic length scales of porosity, as shown in the bottom row of images in Figure 5. This resulted in a ten-fold increase in electric-field-driven ion transport and pressure-driven mass transport through the hierarchical porous network of Au ligaments, compared to those through bulk nanoporous Au without any hierarchical porosity. This technique of homogenizing the sample after printing sidesteps the issue of either making or purchasing costly pre-alloyed powders for additive manufacturing, eliminating one of the more expensive steps in the process.

More recently, Fujita et al. also reported the use of an alternative AM method, selective laser melting (SLM), to make hierarchical porous Cu structures [87]. They were able to purchase commercial Cu-Mn powders for additive manufacturing. Using SLM, they created a series of 3D printed architectures, which were then chemically dealloyed to remove Mn. After dealloying, the result was a nanoporous Cu sample with three scales of porosity: (1) features on the order of hundreds of microns to a few millimeters as defined by the printing process, (2) microscale cracks of 1–5 μm between filaments and (3) a nanoscale porosity with a characteristic pore size of about 140 nm from the selective dissolution of Mn during the chemical dealloying step. These features can be seen in Figure 5a–c. The researchers measured marked improvements in the mass transport and mass-specific catalytic rate, akin to Zhu et al.’s results from hierarchical porous Au. Both reports show the promise of using additive manufacturing to increase the performance of dealloyed materials as highly efficient electrocatalysts and in other energy applications, leading the way in investigating this largely unexplored materials space.

### 4.2. The Matrix of Possibilities for Hierarchical Structures

Both of the previously mentioned studies used chemically dealloying, which is the dealloying method with the longest history and largest body of research, but we anticipate that complementing chemical dealloying with alternative dealloying methods can grow the applications of this technique to include metals beyond the typical noble metals used in chemical dealloying to include metals from nearly every section of the periodic table. Additive manufacturing has been used to construct components across a wide spectrum of compositions, which means dealloying is the limiting factor in the compositional variety of alloys that can be used in this process to create hierarchical porous metals. Electrochemical dealloying itself is fairly versatile, but its composition range is limited by the requirement that the surface mobility of the remaining metal’s atoms fall within a narrow “Goldilocks” range [88]. If the surface diffusivity is too high, the more noble element will passivate the surface, preventing any further dealloying into the bulk. If surface diffusivity is too low, the more noble element will only form clusters as the less noble element is dissolved away, then disconnect from the bulk so the resulting structure is no longer bicontinuous. Liquid metal dealloying—with its broader set of compatible precursor alloys, including the Ti-based alloys and stainless steels—offers materials with better mechanical properties and greater overlap with the structural materials of interest in the AM community. A greater range of materials means a greater range of applications. Solid-state dealloying and vapor phase dealloying further broaden the category of compatible metals. Liquid metal dealloying and solid-state dealloying are resource-intensive, especially compared to vapor phase dealloying, which does not require the use of a dealloying medium or etchant to remove the solidified dealloying medium. Solid-state dealloying and vapor phase dealloying are both recent additions to the growing portfolio of dealloying techniques, and the potential to expand their capabilities to make finer microstructures or work with more material systems has yet to be fully realized. Currently, vapor phase dealloying has only been applied to Zn-based alloys [35,38], which are difficult to incorporate into additive manufacturing due to Zn’s volatility and low boiling point. While the fabrication of hierarchical porous metal structures previously required time-intensive processing, we expect this combined approach will allow the development of these structures to be considerably easier and possible with higher resolutions and greater stability. The field has expended considerable efforts to introduce new AM and dealloying techniques, and the matrix of possible combinations of techniques has grown to allow for immeasurable possibilities for the architectural optimization of hierarchical metal nanostructures. Table 1 lists existing and possible future combinations of dealloying and additive manufacturing techniques based on which processing methods have already been applied to the suitable precursor material.

### 4.3. Metal–Metal Nanocomposites

**Figure 6:**
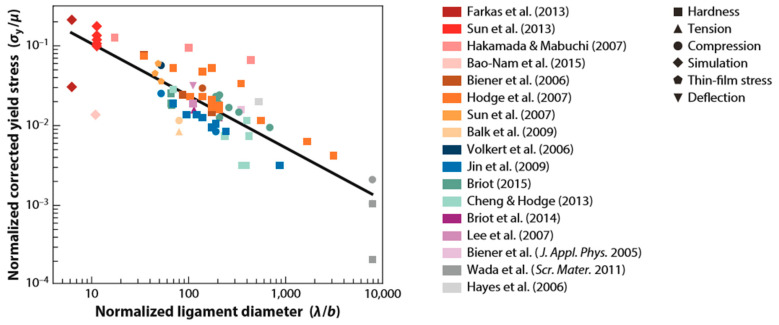
A negative correlation between yield stress and ligament diameter, as shown in these data compiled from various mechanical tests (listed on the right) on nanoporous materials, shows clear evidence of size-dependent strengthening in these materials. Raw yield stress data were corrected to their fully dense yield strength values using their Gibson–Ashby relation and normalized by the material’s shear modulus. Ligament diameter measurements were normalized by the material’s Burgers vector. The black line of best fit gives a power-law exponent very close to power-law values reported for micropillars, which exhibit similar size-strengthening effects. Adapted from [2]. Copyright 2016 by Annual Reviews.

In addition to hierarchical porous structures, additive manufacturing and dealloying together can also enable the fabrication of new nanocomposite materials with improved microstructure and shorter processing times. Here, we discuss the combination of laser-based additive manufacturing—specifically, selective laser sintering/melting (SLS/M)—and dealloying to create metal–metal nanocomposites formed in situ during the melting process. The motivation here is the general observation that materials with small microstructural features generally exhibit higher strength than their bulk counterparts. While selective laser melting itself has been used to fabricate metal composites [75,76], incorporating dealloying produces much finer microstructural features with more complex geometries than disconnected dispersed particles. As the dimensions of the microstructural features decrease, the measured strength of these nanostructures approaches theoretical values [99]. This effect is consistently seen in dealloyed nanoporous metals at a bulk scale; numerous examples are shown in Figure 6. Nanocomposite materials made by liquid metal dealloying (LMD) also show similar behavior, and they incorporate the superior mechanical properties of nanoscale objects into a solid, macroscopic body. In the earliest study of these composites, Wang et al. conducted a mechanical characterization of nanoporous Au impregnated with polymer, reporting improved strength and hardness in the composite material compared to that in each constituent phase [100,101]. More recently, McCue et al. used LMD to produce a series of metal–metal nanocomposites with decreasing average feature sizes and measured dramatic size effects on the mechanical strength of these materials. As they decreased the average feature size in the sample from 10 μm to 70 nm by decreasing the dealloying temperature, McCue and colleagues measured a ten-fold increase in the yield stress of their nanocomposites of interpenetrating Ta/Cu phases [45].

**Figure 7:**
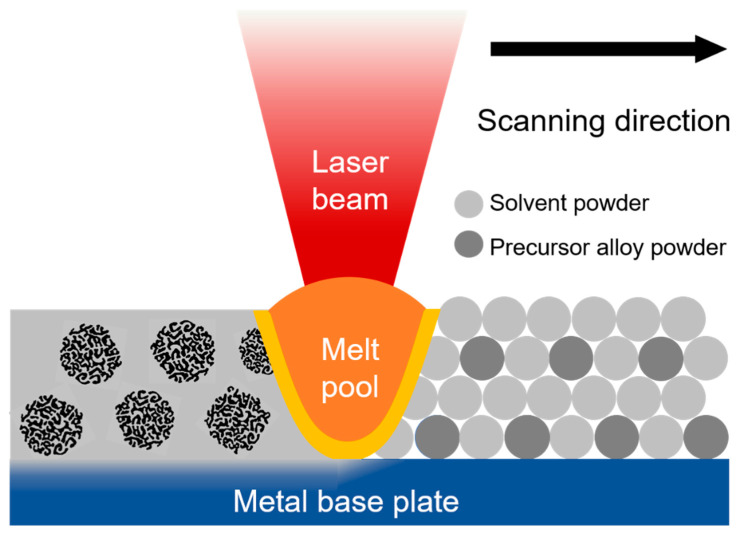
This schematic provides a cross-sectional view of the reactive selective laser melting (SLM) process for printing a 3D composite metal part via in situ dealloying. A melt pool forms where the laser heats the material and acts as small molten bath of liquid metal solvent, in which precursor alloy powders become dealloyed. Once the laser moves on and the material is solidified, the newly fused material is bonded to the base plate and forms a solid composite material with 2 phases: the porous structures of the remaining element from the precursor alloy distributed throughout the solidified solvent phase.

What if a two-component powder mixture was fed into an additive manufacturing system such that dealloying occurred during SLS/M? To our knowledge, this has not been studied in a systematic way. Localized laser interactions with an Ag-Au thin film immersed in nitric acid have been reported as an effective method for patterning the film by selectively dealloying areas irradiated by the laser [102], but introducing the layer-by-layer deposition approach used in AM can scale this process up to 3D geometries. Some work has also been done on dealloying in multi-component compacted powder systems; Zou et al. obtained a bulk sample of bimodal porous copper from plasma-activated sintered powder mixtures of Cu, Fe and Al [103]. However, SLS/M operates on beds of loose powders. Figure 7 illustrates the general concept proposed here. A mixture of dealloying medium metal powder (the “binder”) and the precursor powder is selectively melted with the laser, to the dealloying temperature (above the melting point of the binder), at which point, the binder material melts and the sacrificial element of the precursor dissolves into it, as is usual for LMD. The diameter of the particles is controlled to be larger than the expected dealloyed length scale (~50 nm) but small enough that the particles are still compatible with SLS/M. A powder particle ten microns in diameter would mean that each powder particle would be dealloyed very quickly, assuming bulk LMD dealloying rates on the of order 10 μm/s, as are usually observed. The resultant material would be a 3D printed bulk metal–metal composite.

**Figure 8:**
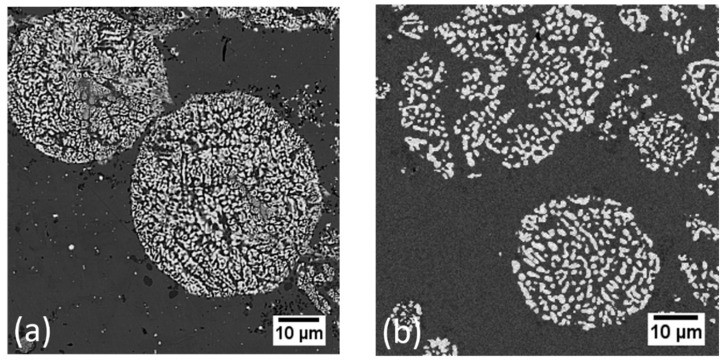
Cross-sectional SEM (scanning electron microscope) images of Ta_30_Ti_70_ powder particles dealloyed by surrounding molten copper powder particles. The lighter phases are the remaining parts of the alloy powders—porous networks of Ta-rich ligaments—after Ti has dissolved out into the copper solvent, and the darker phase is the solidified Cu-rich solvent. Here, we show two different pathways for dealloying powders: (**a**) powders dealloyed by selective laser irradiation, at a scanning speed of 0.75 mm/s, and (**b**) powders dealloyed by heating a compacted powder disk in an induction furnace for two minutes. The second sample shows significant coarsening due to the longer heating time, compared to the more refined microstructure of the powders dealloyed by the laser, which affected how well the particles retained their original shape.

As proof of principle of this concept, Figure 8 shows the cross-section of two powder-based samples, both of which were dealloyed in a mixed powder environment containing Ta-Ti alloy powders mixed together with Cu powders. Once these samples were heated and the Cu powders melted, Ti from the Ta-Ti alloy powders dissolved into the liquid, leaving behind a solid porous network of Ta-rich ligaments. We first conducted this experiment with a compacted powder sample melted in an induction furnace to observe how the dealloying reaction would proceed between powders compared to that in bulk samples. We noted similar relationships between the morphology and processing conditions to those observed in bulk LMD samples and will report details in a later publication. The same powder mixture was then spread in a thin layer over a base plate in a build chamber. Laser-induced heating was effective in melting the Cu powders to induce Ti dissolution and successfully produced one layer of the desired Ta/Cu nanocomposite material. In both cases, Ta-Ti alloy powders, once dealloyed, exhibited the characteristic bicontinuous porosity of LMD materials. We call this concept powder-based dealloying.

The use of multi-component powders also increases processing options from a thermodynamic and phase stability vantage point. For instance, the single element binder can be replaced with powders of a eutectic alloy with a relatively lower melting point, thus lowering the dealloying temperature. Recent work by Song et al. reported dealloying Ta-Ti alloys in molten Cu-based eutectic alloys, yielding a material with a decreased characteristic ligament width of ~75 nm, much finer than Ta-Ti dealloyed in pure Cu, which exhibits ligaments ~3 μm in diameter [104]. However, the dissolving element has decreased solubility in molten eutectic alloys compared to its solubility in single-element solvents, drastically slowing dealloying. McCue et al. discussed how, due to this reduced solubility, they were unable to fully dealloy bulk samples using eutectic alloy baths, only reaching a dealloying depth of 150 μm after 60 min [19] The diffusion limitations in the dealloying medium through the dealloyed region became too severe. In contrast, the diameter of most AM powders is much smaller, so this issue with bulk samples can be also be avoided. Both the dealloying front velocity and the coarsening rate are controlled by Arrhenius kinetics [19], but the activation barriers for liquid phase diffusion are much smaller than the activation barriers for surface diffusion. For this reason, the surface diffusion rate slows by many orders of magnitude more than liquid phase diffusion when the dealloying temperature is decreased. These rates are too slow to make fully dealloyed samples using bulk LMD in eutectic alloys but are suitable for dealloying powders.

Refining our understanding of the integration of dealloying with AM will require a detailed kinetic analysis of the as-formed microstructure in this scenario. We see three kinetic factors that should be characterized: (a) the interface velocity, i.e., how fast the interface is moving between dealloyed and undealloyed material; (b) what the concentration gradient of the dissolving species as it moves through the dissolution medium is; and (c) how fast the microstructure of the dealloyed layer is evolving, i.e., how much coarsening there is behind the dissolution front.

#### 4.3.1. Kinetics of the Interface Velocity

As the molten binder phase becomes saturated with the dissolving species, the driving force for diffusion away from the interface decreases. It is known from bulk LMD kinetic studies that this leads to an interface velocity that slows as *t^1/2^* as a result of the diffusion limitations of the sacrificial element in the molten bath. In the powder bed geometry of AM, the binder has limited mass and volume, in contrast to the functionally infinite bath model considered in bulk LMD, and thus will more quickly approach the solubility limit of the dissolving species in the molten bath. This will likely slow the overall velocity of the dealloying front and eventually stop dealloying altogether. We expect the dealloying process in the mixed powder environment to be limited by the solubility limit of the dissolving element in the binder phase, instead of its diffusion limit, as it is in bulk dealloying systems.

#### 4.3.2. Concentration Gradients in the Dissolution Medium

Approaching the solubility limit may introduce another problem. The remaining component in the precursor is miscible with the dissolving element, so the solubility of the remaining component in the binder phase increases as the dissolving component saturates the binder phase. If the concentration in the binder phase rises to near the solubility limit, some amount of solid porous structure may also dissolve away into the binder. This may have the effect of destroying the bicontinuous porous structure; an equally valid possibility is that the presence of the less-soluble element in the melt could also instead refine the porosity of the solid structure and make the pore size more uniform. Residual quantities of the more noble element of the precursor should precipitate in the binder phase upon cooling and may be observable with SEM/TEM systems.

#### 4.3.3. Coarsening behind the Dissolution Front

Due to the diffusion limitations of the dissolving element diffusing through the liquid medium penetrating the dealloyed layer, the interface velocity decreases, allowing the coarsening of the bicontinuous dealloyed layer to becoming significant. In AM, such secondary coarsening should occur only once a powder particle has been fully dealloyed because the dealloying depth is relatively shallow. Therefore, we can hypothesize that because the dealloying times will be much shorter when dealloying powders, this will lead to smaller ligament sizes. However, if the dealloyed powder remains hot, there is a danger that each dealloyed particle will simply collapse to a ball due to capillary forces, destroying the porosity that was formed. Rapid solidification and fast thermal transport in AM should alleviate this effect. However, another advantage of using powders instead of bulk LMD is that the system can be tuned so that the dissolving species will quickly saturate the molten binder phase, especially if the average binder particle size is small and the molten binder interlayer is thin relative to the powder particle diameter. This should stymie further dissolution from the precursor alloy particles, slowing the dissolution kinetics and extending the working time window.

Porosity evolution can also occur without melting the binder phase via selective laser sintering and solid-state dealloying. In AM, the starting material would be the same two-component powder mixture, but the mixture would be sintered, rather than melted, by the laser-based AM process. The selective laser sintering of the powder mixture induces solid-state dealloying to produce a metal–metal nanocomposite. The dealloying temperature can now fall below the melting point of the binder phase. Widening the operating temperature window broadens the utility of dealloying to include more materials systems [33]. For instance, Ta-Ti dealloyed in Zr is not a viable material system for LMD because Ta has some solubility in Zr above Zr’s melting point, but below 800 °C, the two elements are now immiscible, enabling the formation of a solid porous Ta structure by only dissolving Ti from the precursor and into the solid Zr phase. The dealloying reaction does occur much more slowly, as solid-state diffusion kinetics are orders of magnitude slower. This also lessens coarsening, and the result is a much finer microstructure. Again, while these rates are too slow to make fully dealloyed bulk samples, these slow dealloying front velocities are a good fit for dealloying smaller powder particles.

### 4.4. Challenges Associated with Dealloying Integrated with Laser-Based Processing

The parameters of the sintering or melting process will be critical for the optimization of simultaneous dealloying and solidification. The scan speed, laser power, and melt pool size and shape are only a few of the factors that can be adjusted to improve the quality of the final sample. Previous studies on the additive manufacturing of metal matrix composites can offer insight into additional issues that may arise when processing powders of two different compositions together. Manfredi et al. reported a case study of manufacturing aluminum matrix composites of AlSiMg alloy with two different types of reinforcements, micro-sized SiC particles and nanosized MgAl_2_O_4_ particles [105]. They measured an increase in hardness in the SiC-reinforced alloys compared to that in pure AlSiMg but observed a loss in mechanical properties in the alloys reinforced with MgAl_2_O_4_, which they attributed to changes to the solidification behavior due to the presence of MgAl_2_O_4_ nanoparticles. In a similar study, Vrancken et al. studied the microstructure of Ti_6_Al_4_V powder mixed with pure Mo particles processed via selective laser melting and found local variations in the content of Mo with bands of Mo-rich and Mo-poor compositions [106].

With particle sizes of about 10 microns in diameter and interface velocities on the order of 0.1 microns/s, heating and cooling should be rapid relative to microstructural evolution, but the formation of bicontinuous nanoscale ligaments during dealloying may differ under the extremely fast heating and cooling rates typical in a laser-based additive manufacturing setup, where cooling rates are reported to be between 10^3^ and 10^4^ K/s [107]. Complex thermal processing cycles also complicate the simultaneous fusion of the additive manufacturing process and microstructure evolution of the dealloying process. Much work has already been done to study the effects of repeated melting and solidification in AM samples, which show microstructural banding and evidence of directional solidification [62]. It is well established that the dealloying process generally leaves intact the original crystallographic structure of the precursor material [108], but additive manufacturing could alter the microstructure of the precursor particles and affect the mechanical integrity of the nanocomposite. Post-processing treatments—most commonly, hot isostatic processing (HIP)—have been shown to refine the microstructure in printed parts and mitigate typical SLM/S defects, including residual porosity due to gas entrapment [109]. Many avenues exist for addressing these material challenges, both in changing the AM process itself and developing post-processing techniques to mitigate any mechanical issues that arise from imperfect solidification.

The central question is how refined a microstructure we can expect from an additively manufactured and dealloyed nanocomposite material. We have identified mechanisms that should lead to finer and more homogeneous microstructures: shortened dealloying times, leading to smaller average ligament sizes, and lower operating temperatures with either a eutectic alloy dealloying medium or through solid-state dealloying. With laser-based additive manufacturing, we hypothesize that we can produce metal–metal nanocomposites of nearly any imaginable shape, regardless of complexity, and keep the average length scale of the microstructure uniformly below 100 nm. This would be a huge innovation for materials synthesis via liquid metal dealloying and solid-state dealloying.

## 5. Standalone Porous Powders

**Figure 9:**
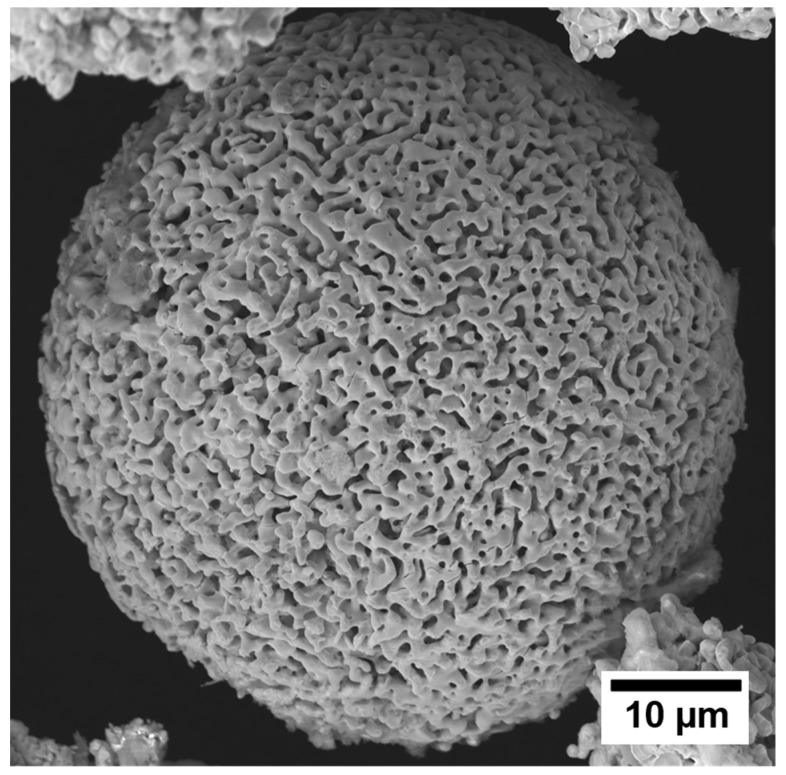
SEM (scanning electron microscope) image of a Ta_30_Ti_70_ powder particle after having been liquid metal dealloyed in copper powder to form a composite and then etched in a dilute HNO_3_ solution to remove the solvent phase. Once the solvent phase is removed, the material is no longer a solid composite and only detached porous alloy powders remain. The porous structure of the Ta-rich particle is bicontinuous, and the particle has retained its original spherical shape.

The materials synthesis approach discussed here can also be used to produce porous powders, which have broad applications in commercial technologies. Nanoporous copper powders demonstrated remarkable catalytic degradation of H_2_O_2_ and elimination of MO in Wang et al.’s experimental investigation into using nanoporous copper powder as a reagent in wastewater treatment [110]. Dealloyed Ti-based alloy powders have also been shown to be effective at removing toxic Cr with excellent adsorption efficiency in aqueous solutions [111]. Figure 9 shows an example of a porous refractory metal powder particle from a compacted two-component powder sample that was liquid metal dealloyed and then immersed in an etchant to dissolve away the binder phase. The metal–metal nanocomposites discussed in the previous section can be etched via immersion in an acidic solution, selectively dissolving away the binder phase, to make standalone nano-/microporous powders. These powders are ideal candidates for applications where the functionality of the material scales with the available surface area. For example, Ta capacitor anodes are made of pressed Ta powders [112]. By replacing the solid powders with dealloyed powders, we can maximize the anodized surface area of these materials. As capacitance scales with surface area, the additional surface area within powders has the potential to greatly improve capacitor performance.

Porous powders can also be backfilled, which presents a unique opportunity to make powders with two interpenetrating bicontinuous phases. Vacuum impregnation is a simple technique to fill the interconnected void space with another material to increase functionality or improve mechanical strength. Bulk liquid metal dealloyed porous refractory metals have been shown to be mechanically robust enough to resist coarsening at temperatures up to 1900 °C [113]. Their high temperature resistance, characteristic of refractory metals, means these samples can be made into composite powders simply by immersing these powders in molten baths of metals with melting points below their own, as long as there is no chemical reaction. This possibility was introduced as a method to tune the mechanical-strength-to-density ratios of composites composed of a dealloyed metallic structure and a lightweight metal [45]. Gaskey et al. presented a proof of concept by backfilling a bulk porous Nb sample with borosilicate glass [113].

### Bulk Porous Structures via Sintering

Porous powders may also serve as feedstock material for AM, enabling them to be “re-formed” into a bulk structure with nanoporosity. This solves a problem with AM, namely, that after dealloying, the porous particles are generally disconnected; see Figure 8. This could be a two-step AM process; first, a powder precursor alloy is dealloyed during a first additive manufacturing process and its binder dissolved to produce a powder, each of whose particles is porous; this material is then used as feedstock in a second AM process to form a bulk 3D part whose shape was dictated by the printing process. As long as the heating power is sufficient to only sinter the particles together and not collapse any porosity, the resulting material should have a fully dealloyed porous structure but with a uniform microstructure that was only possible through the laser-based dealloying of alloy powder particles. These powders can be sintered together in a furnace, but selective laser sintering can offer more control over the design of the final shape.

## 6. Summary and Conclusions

With the recent development of novel dealloying techniques and improvements in additive manufacturing processes, an extraordinary opportunity to design new materials has arrived. A variety of dealloying techniques now allows materials scientists to make nanoporous materials and nanocomposites of nearly any metal on the periodic table and to tailor their shape and microstructure, while the additive manufacturing community has made great strides in printing metal samples that are comparable in mechanical strength and stiffness to their conventionally machined counterparts. Hierarchical metal nanostructures represent one area of interest that can take advantage of progress recently made in both fields. Electrochemical dealloying has already been used to make hierarchical nanoporous materials of additively manufactured bulk components, but liquid metal dealloying or vapor phase dealloying could also be useful techniques for this application. More excitingly, AM plus dealloying promises a one-step synthesis of metal–metal nanocomposites with excellent mechanical properties in any form; the microstructure is formed via liquid metal dealloying or solid-state dealloying, while the overall structure can be nearly any geometry imaginable. These possibilities for new materials come with new processing challenges, and it is clear that further work is necessary to maximize the engineering potential offered by dealloying and additive manufacturing.

## Figures and Tables

**Table 1:** To be used in this combined materials synthesis approach for making hierarchical porous metals, the chosen precursor alloy must be compatible with both AM and dealloying. The elements of the precursor alloy must be chosen such that some material difference (in chemistry, vapor pressure, etc.) between the elements can be exploited for dealloying, but the alloy must also be stable enough to withstand the AM printing process. This table lists alloys that have been successfully printed via AM and dealloyed to produce structures but mostly in separate studies. These potential precursor alloys are categorized by their compatibility with one of two AM processes and one of four dealloying methods. This table represents new opportunities to produce hierarchical nanoporous structures by printing these precursor alloys and then dealloying with a compatible dealloying method.

Compatibility of Precursor Alloys with Dealloying and AM Methods			
		Additive Manufacturing Methods	
		Direct Ink Writing	Selective Laser Melting/Sintering
**Dealloying methods**	**Electrochemical dealloying**	Au-Ag [86], Ni-Cu [14,89]	Cu-Mn [87], Al-Si [90,91]
	**Liquid metal dealloying**	SiC [92,93]	Co-Cr-Mo [28,94], Fe-Cr-Ni [27,95], Nb-Ti [54,96]
	**Solid-state dealloying**	–	Fe-Ni [33,97]
	**Vapor phase dealloying**	–	Brass [37,98]
